# Associations among teacher-child interaction, children's executive function and children's comprehensible vocabulary

**DOI:** 10.3389/fpubh.2023.1077634

**Published:** 2023-03-08

**Authors:** Shi Yan, Min Li, Zhonglian Yan, Biying Hu, Li Zeng, Bo Lv

**Affiliations:** ^1^Faculty of Education, Northeast Normal University, Changchun, Jilin, China; ^2^Faculty of Education, University of Macau, Taipa, Macau SAR, China; ^3^Faculty of Education, Wenzhou University, Wenzhou, Zhejiang, China

**Keywords:** teacher-child interaction, executive function, comprehensible vocabulary, two-way effect, mediating role

## Abstract

**Objective:**

To understand the working mechanism and the relationships among the quality of teacher-child interaction (TCI), children's comprehensible vocabulary (CV) and executive function (EF).

**Methods:**

Using stratified sampling, 900 children (boys 50.2%) and 60 preschool teachers were recruited from 4 places in China for testing, and five measurement tools, including the Classroom Assessment Scoring System (CLASS), the Peabody Picture Vocabulary Test (PPVT-R), the Stroop test, a card sorting task, and the Wechsler Intelligence Scale for Children (WISC-IV), were used.

**Results:**

For every additional unit of TCI, EF increases by 0.55 units; For every additional unit of EF, CV increases by 0.55 units; For every additional unit of CV, EF increases by 0.55 units; For every additional unit of CV, TCI increases by 0.38 units; For every additional unit of TCI, CV increases by 0.38 units. In the Model of TCI-EF-CV, the estimated value of TCI and the total effect of comprehensible vocabulary is 0.18; *Z* = 9.84, which is significantly greater than 1.96 at the bias-corrected 95% confidence interval and at the percentile 95% confidence interval (0.15, 0.23), both of which do not contain 0. The direct effect of TCI and CV is significant and indirect effects account for 39%. In the Model of TCI-CV-EF, the total effect of TCI on executive function is 0.09 (*Z* = 6.14), the direct effect is not significant with bias-corrected 95% confidence interval and 95% confidence interval (−0.01, 0.03), both of which include 0.

**Conclusion:**

There are two-way effects among children's EF and CV, TCI and CV. EF plays a mediating role in the influence of TCI on CV. TCI positively predicts children's EF, but this mainly depends on CV. Therefore, TCI plays a positive role in the development of children's CV and EF.

## Introduction

Language is an important way for children to understand the world (1, 2). Children's vocabulary is the basis of their language experiences (3), and has an important impact on their future academic performance (4–6). The period of 3–6 years of age has been considered as the most important period for children to develop their vocabulary competency (7, 8). According to the ecological theory (9), the development of children's vocabulary in class required the participation of teachers and children. EF reflects the subjectivity of children's participation in their vocabulary development (10).

Early childhood is also a period of rapid development for children's EF (11, 12). EF also has an important impact on children's future academic performance (13–15). According to the theory of dynamic development (16), various cognitive abilities do not develop in isolation, but are interdependent and interactive. In period of 3–6 years of age, vocabulary and EF are related to cognitive ability and should be promoted together (17–19).

TCI have been regarded to benefit the development of children's vocabulary and EF (20–22), Even TCI based on children's vocabulary and EF can allow teachers to guide children in a more targeted manner (23).

Few studies have focused on the interactions among EF, vocabulary and TCI. Therefore, based on ecosystem theory, this study aimed to examine the influence of TCI and EF on vocabulary; this will not only help clarify the research on the role of teacher and child factors in children's vocabulary but also provide directions for subsequent interventions for children's vocabulary and the exploration of new ways to improve the quality of early childhood education by TCI.

### The effect of executive function on comprehensive vocabulary

EF is a series of top-down mental processes that occurs when children need to concentrate and pay attention; these processes involve three core components, including working memory, cognitive flexibility, and inhibitory control (24, 25), which together are termed EF. The period of 3–6 years age is a period of rapid EF development in children (11, 12).

At the same time, children's vocabulary in this period also develops rapidly (7). Vocabulary is the basis of children's language acquisition (3) and is divided into receptive vocabulary and expressive vocabulary.

Comprehensive vocabulary is receptive vocabulary that can be understood in receptive language activities such as listening and reading (26). During period of 3–6 years of age, comprehensive vocabulary regular develops better and earlier than expressive vocabulary (27).

Researchers found that EF is related to children's vocabulary (28), and even has a positive predictive effect on children's vocabulary (17–19), which is mainly reflected in the function of inhibition control (4).

The understanding and use of vocabulary require attention, reasoning, integration and working memory. Children with high level of EF can process information faster (29), EF skills can help children focus on multiple streams of information at the same time, monitor errors, and make decisions based on available information, which can help them improve their vocabulary level (24).

### The influence of TCI on comprehensive vocabulary and EF

TCI is the teacher's response to the behavior that children are engaged in (30–32). TCI affects children's development through three aspects: emotional support, class organization, and guidance support (33). According to attachment theory (34), children's development is greatly influenced by their emotions. In kindergarten, emotional support is mainly reflected in the warm relationship between teachers and children that is conducive to children's learning and development (35, 36), and emotional support is considered to be related to children's social development [(37, 38), p. 49–84].

From the perspective of ecological theory (9), in the classroom ecological system in which teachers and young children coexist, effective teaching support is reflected in teachers' effective strategies (such as scaffolding and brainstorming) to support children's thinking (23). Studies have also found that the higher the teacher's guidance and support in the class is, the greater the improvement of children's preacademic skills (39).

The quality of early childhood education is closely related to children's development and TCI (40, 41). In class ecology, high-quality TCI involving children's development is common; that is, TCI is closely related to EF and children's language (42, 43).

**Figure 1:**
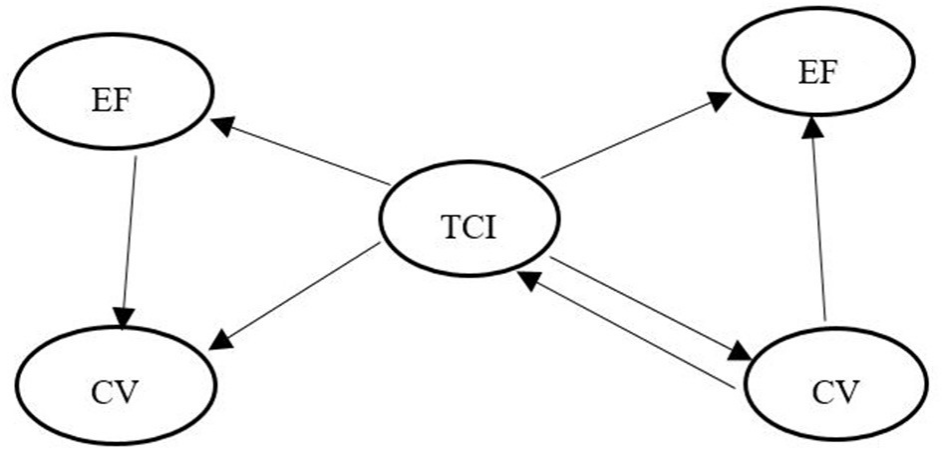
The hypothesis-diagram of-three-variable relationship.

High-quality TCI helps improve children's EF levels (20, 21, 44), not only due to positive emotional support but also to the effective educational support provided by teachers (44), especially through the improvement of teachers' behavior, which is conducive to the development of children's cognitive development and self-control. Since the development of children's EF is an internalization process of children's spontaneous completion of concepts (45), the interaction between teachers and children is conducive to children's completion of this internalization process (46). This means that the development of EF can be achieved through activities organized by teachers (47). Since teachers' management of activities helps children's self-restraint (48), and peer relationship affects children's learning enthusiasm, high-quality TCI is generally conducive to increasing children's enthusiasm for participation and realizing children's self-inhibition and restraint. Therefore, hypothesis H1 is proposed: TCI positively predicts children's EF (Figure 1).

TCI can promote children's language development (49, 50) and even vocabulary development (51). Researchers have found that high-quality TCI helps children learn vocabulary (52, 53). That is, the higher the quality of TCI (such as teachers' emotional and cognitive abilities) is, the higher the level of children's vocabulary development in the early stage. The main reason is that high-quality TCI can provide children with positive emotional support. Therefore, researchers believe that high-quality TCI has a predictive effect on children's CV (54, 55). Researchers even believe that the quality of TCI can positively predict the amount of vocabulary acquisition by children (56, 57).

### The influence of comprehensive vocabulary on EF and TCI

From the perspective of symbolic communication theory, TCI is communication interaction with the help of language symbols (such as words, expression, posture and other symbols) (20, 58). Researchers found that the growth of vocabulary can increase the level of EF (59, 60), due to the relationship between vocabulary and cognition. Children's internal and external languages help improve children's EF skills (61, 62). Before the generation of advanced psychological functions (such as EF), private speech in young children precedes their thinking and is the carrier of thinking, and the generation process is also the thinking process (45, 63).

According to the theory of dynamic development (16), the essence of children's development is dynamic, various cognitive abilities are not developed in isolation, but are interdependent and interactive. In the period of 3–6 years of age, vocabulary and EF should be promoted together (17–19). The relationship between EF and children's language ability is two-way (64–66). Researchers have found that children's comprehensive vocabulary (CV) and EF predict each other (60). Inhibition control and attention shift promoted the two-way prediction of EF and CV as shown through experiments (66). Therefore, hypothesis H2 is proposed: there is a two-way influence between EF and children's CV (Figure 1).

Children's vocabulary plays an important role in children's language development (3). Only TCI based on children's vocabulary can allow teachers' guidance to children to be more targeted (23), and in this way, teachers can provide more instructive scaffolding to support children's vocabulary development, especially to mobilize children's learning motivation, interest, learning strategies, etc. [(67), p. 149–162]. Therefore, children's vocabulary level also has a certain impact on TCI.

In summary, the relationship between TCI and CV is as follows: on the one hand, TCI affects CV; on the other hand, CV affects TCI. Therefore, hypothesis H3 is proposed: there is interaction between CV and TCI (Figure 1).

### EF as a moderator

Since TCI can positively predict CV (51, 54, 55), EF can positively predict CV (17–19), and TCI can positively predict EF (21, 44). EF mediates the relationship between parents' behavior and children's behavior (68–71), EF also plays an intermediary role between variables at the individual level, such as the intermediary role between health and academic achievement (72), early education experience and academic achievement (73), emotion and academic achievement (74).

In addition, children's EF is developing rapidly in early childhood (75), and relevant factors may affect the development of EF (68, 69, 73, 74), the working memory and inhibition control of EF are involved in their own behaviors (68, 69, 71, 73), which ensures the mediation of EF. Therefore, hypothesis H4 is proposed: children's EF mediates the influence of TCI on CV (Figure 1).

### CV as a moderator

Since TCI can positively predict EF (21, 44), it positively predicts CV (51, 54, 55), and CV positively predicts EF (59, 60). Therefore, it is possible that CV mediates in the influence of TCI on EF.

The researchers found that children's language mediates their own development by external factors (76, 77) and plays an intermediary role between different variables at the individual level (78, 79). As the basis of language (3), vocabulary may participate in or hold the intermediary role of language.

In fact, vocabulary mediates the development of external factors and individual abilities (80, 81) since external factors (such as mother's language and socio-economic status) positively predict children's vocabulary levels, and vocabulary level positively predicts a kind of ability (such as EF). Thus, vocabulary may mediate the relationship between external factors and EF (80, 81). Therefore, hypothesis H5 is proposed: Children's CV mediates the influence of TCI on children's EF (Figure 1).

### TCI as a moderator

Since CV positively predicts EF (59, 60), TCI also positively predicts EF (20), CV also affect TCI (82). TCI may mediate the relationship between CV and EF.

In fact, some researchers have also found that TCI plays a mediating role, Hu et al. (82) found that TCI mediates the relationship between preschool education investment and children's academic achievements. Shim and Lim (83) found that TCI mediates the impact of teachers' work environment and self-efficacy on children's peer play since external factors impact the quality of TCI, which in turn affects the development of children (82, 83). Therefore, hypothesis H6 is proposed: TCI mediates the influence of CV on EF (Figure 1).

## Materials and methods

### Participants

According to the Ethics Committee of the first author's university, after obtaining the authorization of the participants (the kindergarten principal, kindergarten teachers and parents), 3 public kindergartens (one high-quality, one medium-quality, and one qualified) and 2 private kindergartens (1 high-quality kindergarten and 1 medium-quality) were selected randomly in Changchun City and Yongji County in Jilin Province and Datong City and Hunyuan County in Shanxi Province by stratified sampling. Sixty teachers and 900 young children from a total of 20 kindergartens in four places were the research objects. In each kindergarten, 3 classes (one senior, middle and young) were randomly selected, for a total of 60 classes; there was 1 teacher in each class, for a total of 60 teachers, and 15 children in each class. The 15 children were volunteered by their parents and recommended by the teacher in the class (Table 1). There was one-to-one correspondence between the teachers and the children.

**Table 1:** Demographic distribution of research objects.

	**Category**	**Quantity**	**Percentage**
Child's sex	Boy	471	52.3%
	Girl	429	47.7%
Class	Young	300	33.3%
	Middle	300	33.3%
	Senior	300	33.3%
Teacher's age	25 years old and below	17	28.3%
	26–39 years old	40	66.7%
	Over 40 years old	3	5.0%
Teaching tenure	Within 5 years	22	36.7%
	More than 6 years	38	63.3%
Teacher education	College	20	33.3%
	Bachelor's degree and above	40	66.7%
Teacher title	No title	45	75.0%
	Title	15	25.0%
Location	City	405	45.0%
	Township	495	55.0%
Type of kindergarten	Private	585	65.0%
	Public	315	35.0%

### Measures

Based on the Ethics Committee of the first author's university, after collecting the literature, the researchers determined the research plan. The selected research tools were authorized.

Teacher-child interaction was assessed using the CLASS developed by the team led by Pianta et al. (84); The CLASS system is fully introduced on the website (www.teachstone.org). The video analysts in this study were trained in the CLASS system. The scale is divided into three dimensions, namely, Emotional Support, Classroom Organization, and Instructional Support, with 10 subdimensions and 42 evaluation indicators (85). The Cronbach's alpha coefficients were between 0.73-0.813, the fit between the three variables was acceptable (χ^2^/df = 1.351, RMSEA = 0.077, CFI = 0.967, TLI = 0.953, RFI = 0.841, NFI = 0.887, IFI = 0.968), indicating that the tools to measure TCI were credible.

The CV level was assessed using the Peabody Picture Vocabulary Test (PPVT-R) [revised by (86)]. The full set of PPVT-R tests has a total of 123 pictures (3 examples, 120 items), each of which has four black and white line drawings suitable for children from 3 years and 3 months to 8 years and 5 months. In the formal test, the tester speaks a word and asks the children to point out a picture that matches the meaning of the word. The correct answer is 1 point; otherwise, it is 0 points. If there are 6 errors in 8 consecutive questions, the test is ended. To determine the child's test score, the ordinal numbers of the previous wrong questions are subtracted from the ordinal number of the last picture answered by the child, and the score ranges from 0 to 120 points. The Cronbach's alpha was >0.831, indicating that the tools used to assess narrative ability were credible. The research tool was introduced in the paper by Sang and Miao (86).

According to the current research results of scholars on EF, the EF test in this study included three parts: inhibitory control, cognitive flexibility, and working memory. Among them, suppression control was assessed using the day/night Stroop task tool (87, 88), and the Cronbach's alpha was 0.744. Cognitive flexibility was measured using a card sorting task tool designed by Frye and adapted by Fei et al. (89, 90), and its Cronbach's alpha was 0.960. Working memory was assessed using the back-to-back digital test tool (91) in the Wechsler Intelligence Scale for Children (WISC-IV), and its Cronbach's alpha was 0.933, indicating that the tools of the three dimensions of EF could be trusted. The two research tools were specifically introduced in the papers published by Fei et al. (89) and Gai et al. (91).

### Procedure

First, we have established a theoretical framework for this research and built a complex mediation model.

Second, according to the ethical requirements of the Faculty of Education of Northeast Normal University, the kindergarten head, parents of the children participating in the research, and teachers were informed of the research content and signed the research ethics specification in class.

Third, the post-graduate pre-school education students who participated in the survey were trained for 3 days by personnel who had participated in training and administered the survey, the training included information on the relevant ethical and technical requirements, and the trainees performed on-site technical demonstrations to ensure that they met the requirements.

Fourth, after the testers arrived at the kindergartens, the teacher in each class provided a list of participants, and the testers conducted a CV test and a test of EF. The researchers tested the children in four counties in China.

Fifth, the teacher provided a class activity video. After the uniform number, the video was analyzed by the researcher who received the CLASS training. The data analysts who participated in this study received training in the CLASS system.

Sixth, there is a one-to-one correspondence between the video analysis results and the children's test results.

### Data analysis

To ensure that this study conforms to ethical requirements, we obtained the consent of the parents when conducting the test, emphasized that the test was voluntary, and ensured the anonymity and confidentiality of the information collected.

**Figure 2:**
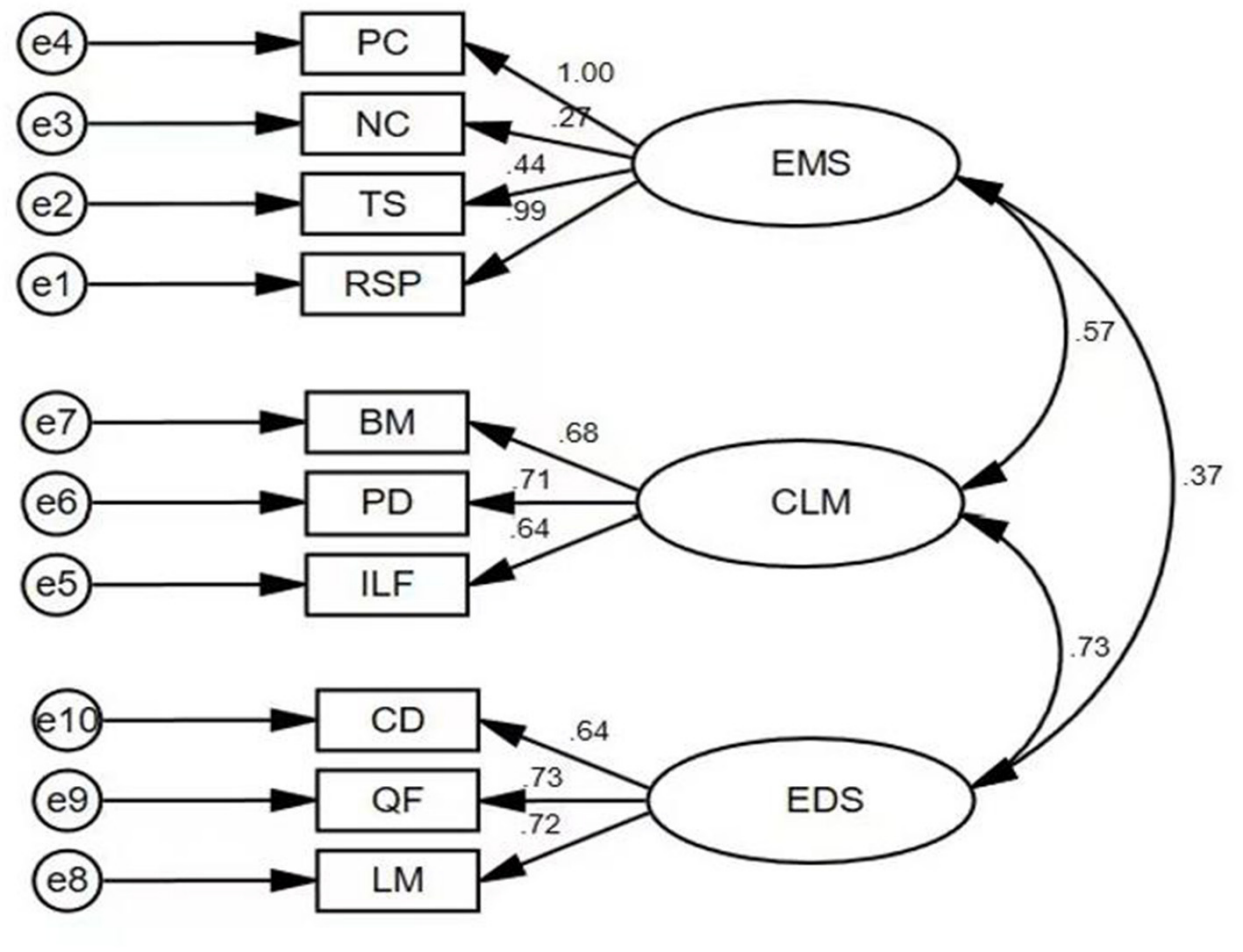
Factor loadings of the CLASS. ES, Emotional support; CM, Class management; ES, Educational support.

To ensure the cultural adaptability of the CLASS tool, we performed a confirmatory factor analysis. The study used 60 teachers' TCI scores for confirmatory factor analysis (Figure 2). Further analysis of the degree of fit (Table 2) showed that the model had a good degree of fit, indicating that the CLASS evaluation system had good structural validity [(92), p. 41–53]. With the help of Amos 23.0, the analysis of fit indices was carried out on the three variables, CV, EF, and TCI: X2/df = 4.101 < 5, RMR = 0.012 < 0.05, RMSEA = 0.080 ≤ 0.08, GFI, TLI >0.08, and NFI, IFI, and CFI were all >0.09, indicating that the fit between the three variables was acceptable [(92), p. 41–53].

**Table 2:** Correlation matrix of TCI, CV, and EF.

	***M***	**SD**	**1**	**2**	**3**	**4**	**5**
1. TCI	5.21	0.58	-				
2. CV	43.78	23.83	0.38^**^	-			
3. Inhibition control	0.35	0.19	0.16^**^	0.42^**^	-		
4. Working memory	0.14	0.13	0.19^**^	0.40^**^	0.53^**^	-	
5. Cognitive flexibility	2.18	0.82	0.22^**^	0.50^**^	0.55^**^	0.59^**^	-
6. EF	0.89	0.34	0.23^**^	0.55^**^	0.69^**^	0.69^**^	0.98^**^

^**^p < 0.01.

TCI, teacher-child interaction; CV, comprehensible vocabulary; EF, executive function.

Finally, after eliminating invalid data, this study used SPSS 26.0 to carry out the deviation test of common methods, descriptive statistical analysis of variables, correlation analysis and regression analysis. AMOS23.0 was used to test the structural equation model and perform the Bootstrap analysis. The Bootstrap method takes 5,000 samples and estimates the 95% confidence interval.

## Results

### Correlation

The linear correlation analysis found (Table 2) that TCI and EF are significantly positively correlated, with a correlation coefficient of 0.23 (*p* < 0.01). Specifically, the correlation coefficient between TCI and inhibition control is 0.16, the correlation coefficient with working memory is 0.19, and the correlation coefficient with cognitive flexibility is 0.22. TCI is significantly positively correlated with CV, with a correlation coefficient of 0.38 (*p* < 0.01). CV has a significant positive correlation with EF, with a correlation coefficient of 0.55 (*p* < 0.01). Specifically, the correlation coefficient between CV and inhibition control is 0.42, the correlation coefficient 0.19, and the correlation coefficient with cognitive flexibility is 0.50.

### Regression analysis

Under the premise that TCI is significantly positively correlated with children's EF, the interpretation and prediction of TCI on children's EF can be further tested. In this study, TCI was used as the independent variable, and the children's EF was used as the dependent variable. The forced input method was used to carry out linear regression analysis. In the regression model of TCI and children's EF, the adjusted *R*^2^ value is 0.30 (Table 3), indicating that TCI predicts 30% of the children's EF. The standardized regression coefficient β value and the significance level Sig value show that TCI has an extremely significant positive predictive effect on children's EF and that every additional unit of TCI increases the EF by 0.55 units, which means hypothesis H1 is partially supported.

**Table 3:** Regression model among TCI, CV, and EF.

**Dependent variable**	**Independent variable**	***B***	**SE**	**Adjusted R^2^**	**Δ*R*^2^**	**β**	**Sig**
CV	TCI	0.15	0.01	0.14	0.14	0.38	0.000
EF	TCI	0.18	0.2	0.05	0.05	0.23	0.000
CV	EF	0.32	0.02	0.30	0.30	0.55	0.000
TCI	CV	0.97	0.08	0.14	0.14	0.38	0.000
EF	CV	0.94	0.05	0.30	0.30	0.55	0.000

Under the premise that EF and CV are significantly positively correlated, the interpretation and prediction of CV by EF are further tested. Using the above method for analysis, it is found that EF predicts 30% of children's CV. From the standardized regression coefficient β value and the significance level Sig value, it can be seen that EF has an extremely significant positive predictive effect on children's CV and that every additional unit of EF increases the CV by 0.55 units, which means hypothesis H2 is partially supported.

Under the premise that children's CV is significantly positively correlated with children's EF, the interpretation and prediction of children's CV on their EF can be further tested. Using the above method for analysis, it is found that the predictive power of children's CV on EF reaches 30%. For every additional unit of children's CV, EF increases by 0.55 units (Table 3); that is, there is a bidirectional predictive relationship between EF and CV, which is similar to the results of existing research (64, 65), which means hypothesis H2 is supported.

Under the premise that TCI is significantly positively correlated with children's CV, the interpretation and prediction of TCI can be further tested. Similarly, using the above method for analysis, it is found that the predictive power of CV for TCI reaches 14%. For every additional unit of children's CV, TCI increases by 0.38 units, which means hypothesis H3 is partially supported.

Using the above method for analysis, it is found that the predictive power of TCI for CV reaches 14% (Table 3). From the standardized regression coefficient β value and the significance level Sig value, it can be seen that TCI has an extremely significant positive predictive effect on children's CV and that every additional unit of TCI increases the CV by 0.38 units, which means hypothesis H3 is supported.

### Test of the mediating effect

The regression analysis results between the three variables can be used for model construction. With the help of Amos 23.0, three models were constructed with TCI, EF, and CV as dependent variables, independent variables, and intermediate variables. According to the operating procedure, the sample size was set to 5,000, the confidence interval was 95%, and the calculation was performed. After analysis, the functional mediation model fit the data well [(92), p. 41–53]. The mediation effect analysis results are as follows.

**Figure 3:**
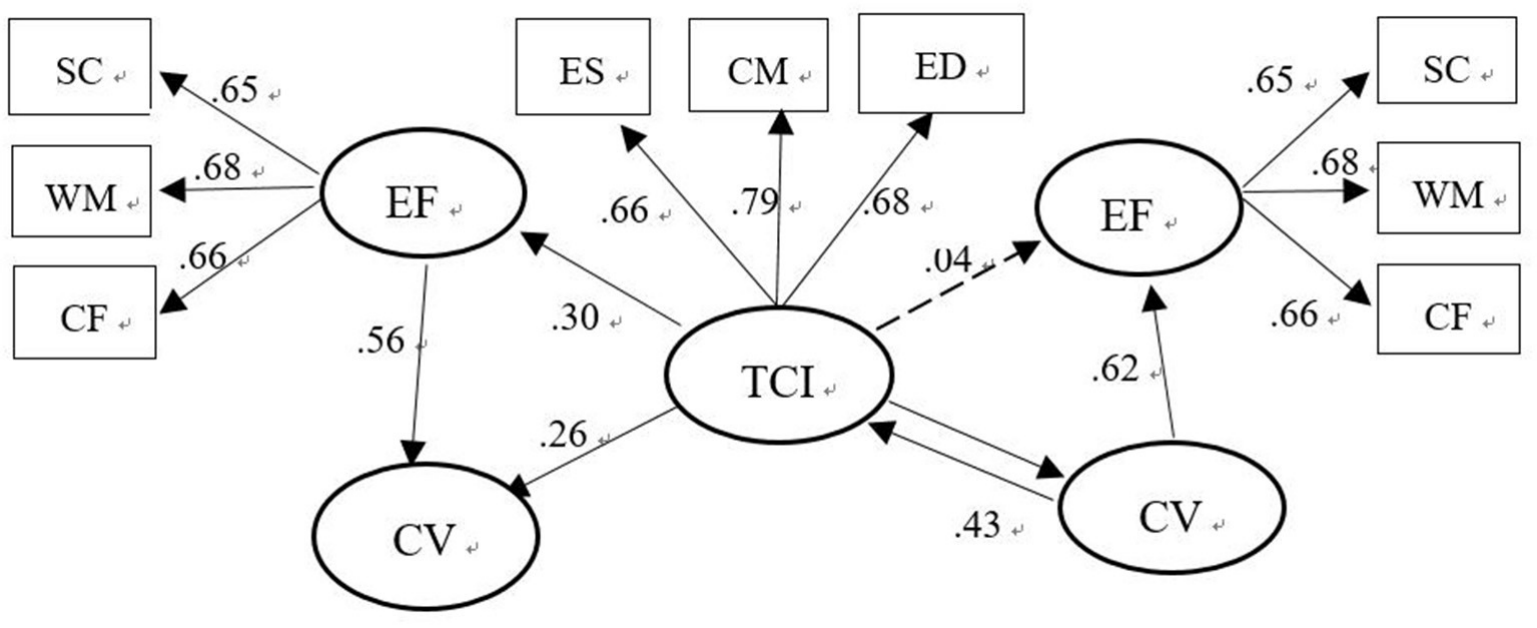
The model of three-variable relationship.

In Model 1 (Table 4 and Figure 3), that is, in the path (TCI- CV), TCI is the independent variable, CV is the dependent variable, and EF is the mediating variable. The estimated value of TCI and the total effect of CV is 0.18; Z = 9.84, which is significant and >1.96, and the bias-corrected 95% confidence interval and the 95% confidence interval (0.15, 0.23) do not contain 0, indicating that the total effect of TCI and CV is significant. In the same type of analysis, it is found that EF has significant indirect effects on TCI and CV and that the direct effect of TCI and CV is significant, of which indirect effects account for 39%. In summary, EF plays a mediating role in the influence of TCI on CV, which means hypothesis H4 is supported.

**Table 4:** Bootstrap test of mediation.

**Model**	**variable**	**Effect**	**Point estimate**	**Product of coefficient**		**Bootstrapping**			
						**Bias-corrected 95% CI**		**Percentile 95% CI**	
				**SE**	***Z***	**Lower**	**Upper**	**Lower**	**Upper**
M1		Total	0.18	0.02	9.84	0.15	0.23	0.15	0.23
	TCI-CV	Indirect	0.07	0.01	6.08	0.06	0.10	0.05	0.10
		Direct	0.11	0.01	7.60	0.09	0.14	0.09	0.15
M 2		Total	0.09	0.01	6.14	0.06	0.09	0.06	0.12
	TCI-EF	Indirect	0.08	0.01	8.44	0.06	0.09	0.06	0.09
		Direct	0.01	0.01	0.83	−0.01	0.03	−0.01	0.03
M 3		Total	0.42	0.03	16.60	0.37	0.47	0.37	0.47
	CV-EF	Indirect	0.01	0.01	0.83	−0.01	0.03	−0.01	0.03
		Direct	0.41	0.03	13.97	0.35	0.46	0.35	0.46

In Model 2, that is, in the path from TCI to EF, the total effect of TCI on EF is significant, which mainly occurs through indirect effects. The direct effect with a bias-corrected 95% confidence interval and a 95% confidence interval of (−0.01, 0.03) include 0, so the direct effect is not significant (Table 4 and Figure 3). That is, the influence of TCI on children's EF is mainly completed through children's CV. CV play a mediating role between TCI and children's EF, which means hypothesis H5 is supported.

In Model 3, that is, in the path from CV to EF, the overall effect of CV on EF is significant, and it mainly occurs through direct effects with bias-corrected 95% confidence interval and percentile 95% confidence intervals (−0.01, 0.03) for indirect effects contain 0, while the indirect effects are not significant (Table 4). TCI did not mediate the effect of CV on EF, which means hypothesis H6 is not supported.

## Discussion and analysis

### There is bidirectional influence between children's EF and CV

Children's EF affects children's CV, mainly reflected in the function of EF. Generally, children with a high memory level, flexible cognition and strong inhibition ability are better at learning, children with high EF level are better at learning, have higher memory level, and have stronger ability to control irrelevant factors to interfere with learning, that is, executive function positively predicts children's CV (17, 19).

Children's EF and CV are interdependent and interactive, which provides empirical evidence for the dynamic development view (16) and supports recent research results on the two-way relationship between EF and CV (64–66). The dimensions of EF are related to CV (Table 2), especially the function of inhibition control and attention shift, which improves children's vocabulary learning efficiency (66). The higher the level of EF is, the faster the children can process information (29).

The higher the level of children's inhibition control and working memory, the more individuals can focus, inhibit the interference of irrelevant information, pay attention to multiple information sources at the same time, and make decisions on the basis of existing information (24), allowing children to make full use of language learning opportunities to improve their language ability.

Preschool children's CV also plays a role in promoting EF which may be the reason that language, as an important symbolic intermediary, gradually internalizes from initially externally oriented language in the process of development to form internal language, which can play a role in reflecting and guiding external behavior in the process of solving problems so that children perform better in completing EF tasks (45).

The direct influence of CV on EF is determined by the development mechanism of thinking itself. Because CV plays an important role in children's language development, it is a tool for children's thinking and communication (27). In Vygotsky's view, children's language is closely related to children's thinking (45). Children have a high level of CV, a high level of language, a high level of thinking, a high level of inhibition and control, and a high level of working memory, which is also conducive to the development of children's cognitive flexibility, which means the level of children's EF is also high. On the other hand, the higher the level of children's CV, the easier it is for children to understand rules (21), improve their inhibition ability, and improve their EF.

The combination of these two aspects shows that the influence between CV and children's EFF is bidirectional, which has been consistent with research conclusions (64, 65).

### There is bidirectional influence between TCI and CV

On the one hand, children's CV can positively predict the quality of TCI. TCI is based on language, and children are also participants in TCI. Children with a good vocabulary comprehension level are better at expressing (26), which is more conducive to children's understanding of the language information sent by teachers in the process of TCI, and they are also better at expressing information to teachers. That is, children's CV level can positively predict the quality of TCI, which is the same as the existing research conclusions (64).

On the other hand, the quality of TCI can positively predict children's CV. Children's language development is one of the contents of preschool education and one of the aims of TCI. High quality TCI can provide children with positive emotional support, which is very conducive to improving children's motivation to participate in activities (52, 53). Therefore, it should promote children's language development, including children's CV (49–51).

The combination of these two aspects shows that the influence between TCI and children's CV is two-way, which is consistent with the existing research conclusions (64, 65).

On the one hand, TCI positively predicts children's CV. From the perspective of attachment theory (93, 94), children have emotional needs (95), and high-quality TCI includes providing children with behavior guidance based on attachment (85, 96), teachers' emotional support helps motivate children to learn vocabulary (93, 97).

On the other hand, children's CV also positively predicts TCI, and TCI is the core driving force of children's learning (98). Teachers and children interact with each other through words, expressions, posture and other symbols and interact with meaningful symbols such as pictures, photos and videos. Children achieve active development during their interactions with teachers, promoting cognitive ability, self-control ability, EF and language (20, 58). Children with higher vocabulary level are more conducive to the communication between themselves and teachers, and their quality of TCI will be higher.

Generally, high-quality teachers' TCI may be based on children's experience (99). Children with strong vocabulary ability are more conducive to teachers' adoption of different strategies. From the perspective of ecological theory (9), in children's classes, children's individual characteristics should be the initial basis for TCI (100). During the TCI with the help of vocabulary communication, children with a high vocabulary level can also better understand the guidance of teachers (85).

### Children's EF plays a mediating role in the influence of TCI on CV

According to the ecological theory (9), children's language development requires the participation of both teachers and children. TCI is an external factor in the development of children's CV, while EF is an internal factor. As children are relatively young, they need more emotional support from adults. Emotional support in children's vocabulary learning can be achieved through TCI. In fact, the direct impact of TCI on children's CV learning accounts for 87.62% of the total (Table 3). Teachers can also influence the children's EF through TCI, guide children to strengthen self-control, improve learning strategies (67), and provide targeted guidance (82), thereby improving the level of CV learning.

On the one hand, TCI has a positive predictive EF. In a class directed at children's vocabulary learning, teachers in Chinese cultural contexts should pay attention to class management. From the perspective of Vygotsky's developmental theory, children's learning is related to their surrounding lives. In fact, class management enables children to learn self-control. In class, interactions with teachers and peers helps children learn rules and self-control (45, 94), which shows that the communication between teachers and children is conducive to the development of children's EF (101).

On the other hand, children's vocabulary learning is the result of the participation of children, teacher and peers. Children's vocabulary develops through the process of dynamic adaptation between teachers and children and the process of mutual promotion between children's internal EF and vocabulary (99). In class ecology (9), teachers' targeted guidance and the peer communication created by teachers are also conducive to children's vocabulary learning. Self-discipline and self-control of EF effectively promote children's enthusiasm and effectiveness in vocabulary learning (24).

### Children's CV mediates the influence of TCI on EF

The intermediary function of vocabulary in the relationship between TCI and EF is essentially the embodiment of TCI due to the role of TCI in promoting vocabulary and the role of vocabulary in thinking, and the role of vocabulary in thinking particularly reflects EF. From the perspective of symbolic communication theory, interpersonal communication is carried out by means of symbols, and vocabulary is the symbol of interaction and communication between children and teachers (20, 58). At the same time, during TCI, vocabulary also plays a role in thinking, especially in the transformation of external vocabulary into internal language, which plays a guiding role in solving problems and well implements the task of EF (45).

## Conclusion and suggestions

### Conclusion

There is a two-way influence between children's CV and EF, and between TCI and children's CV. Children's CV can positively predict EF, and EF can also positively predict children's CV; The quality of TCI positively predicts children's CV, and CV can also positively predict TCI.

EF plays a mediating role in the influence of TCI on CV. The influence of TCI on CV is through the direct influence of TCI on CV, and also through the indirect effect of EF on CV.

TCI positively predicts children's EF, but this is mainly achieved through the indirect role of CV. TCI has little direct impact on EF.

### Suggestions

Preschool is a critical period for children's vocabulary development (7), and language also affects other aspects of children's development. According to the development level of children, selecting the content that is suitable for children's life, especially the content related to children's CV, is conducive to mobilizing the enthusiasm of children's participation and achieving the dual promotion of children's CV and EF. The improvement of children's CV is also conducive to the improvement of the quality of TCI. In this way, children and teachers can promote each other and achieve mutual sustainable development by guiding children's development and improving preschool teachers' educational ability.

Therefore, kindergarten teachers should take the development of children's CV as the starting point, implement effective TCI, and then realize the common development of children's EF, CV and the quality of TCI.

### Implications

First, the purpose of the acquisition of CV is to promote the development of children's narrative ability, and the effect of CV on the development of children's narrative ability remains to be further studied. Second, the quality of TCI is considered to be related to the development of children (39, 85) but not related to social communication skills (23), which needs to be further studied. Second, the effect of TCI on children may be revealed to be better and more convincing by longitudinal follow-up research, which also requires further research.

## Data availability statement

The original contributions presented in the study are included in the article/supplementary material, further inquiries can be directed to the corresponding author.

## Ethics statement

This study was carried out following the recommendations of the Declaration of Helsinki (2013) and the Ethics Review Board of Northeast Normal University.

## Author contributions

Conceptualization and writing—review and editing: ZY and SY. Methodology and visualization: BH and ZY. Software: ML. Validation: ZY, SY, and BH. Formal analysis: ZY and ML. Investigation: SY and ML. Data curation: ML, LZ, and BL. Writing—original draft preparation: ZY, SY, and ML. Supervision: ZY, BH, and BL. Project administration and funding acquisition: ZY. All authors have read and agreed to the published version of the manuscript.
